# A Vision-Based Driver Nighttime Assistance and Surveillance System Based on Intelligent Image Sensing Techniques and a Heterogamous Dual-Core Embedded System Architecture

**DOI:** 10.3390/s120302373

**Published:** 2012-02-23

**Authors:** Yen-Lin Chen, Hsin-Han Chiang, Chuan-Yen Chiang, Chuan-Ming Liu, Shyan-Ming Yuan, Jenq-Haur Wang

**Affiliations:** 1 Department of Computer Science and Information Engineering, National Taipei University of Technology, 1, Sec. 3, Chung-hsiao E. Rd., Taipei 10608, Taiwan; E-Mails: ylchen@csie.ntut.edu.tw (Y.-L.C.); cmliu@csie.ntut.edu.tw (C.-M.L.); 2 Department of Electrical Engineering, Fu Jen Catholic University, New Taipei City 24205, Taiwan; E-Mail: hsinhan@ee.fju.edu.tw; 3 Department of Computer Science, National Chiao Tung University, 1001 University Road, Hsinchu 30050, Taiwan; E-Mails: gmuooo@gmail.com (C.-Y.C.); smyuan@gmail.com (S.-M.Y.)

**Keywords:** CCD sensors, computer vision techniques, driver assistance systems, embedded systems, heterogeneous multi-core systems, nighttime driving

## Abstract

This study proposes a vision-based intelligent nighttime driver assistance and surveillance system (VIDASS system) implemented by a set of embedded software components and modules, and integrates these modules to accomplish a component-based system framework on an embedded heterogamous dual-core platform. Therefore, this study develops and implements computer vision and sensing techniques of nighttime vehicle detection, collision warning determination, and traffic event recording. The proposed system processes the road-scene frames in front of the host car captured from CCD sensors mounted on the host vehicle. These vision-based sensing and processing technologies are integrated and implemented on an ARM-DSP heterogamous dual-core embedded platform. Peripheral devices, including image grabbing devices, communication modules, and other in-vehicle control devices, are also integrated to form an in-vehicle-embedded vision-based nighttime driver assistance and surveillance system.

## 1. Introduction

Traffic accidents have become a major cause of death. Most traffic accidents are caused by driver carelessness under traffic conditions. Therefore, detecting on-road traffic conditions for assisting drivers is a promising approach to help drivers take safe driving precautions. Accordingly, many studies have developed valuable driver assistance techniques for detecting and recognizing on-road traffic objects, including lane markings, vehicles, raindrops, and other obstacles. The objects are recognized from images of road environments outside the host car [1,2]. These driver assistance techniques are mostly developed based on camera-assisted systems, and can help drivers perceive possible dangers on the road or automatically control the apparatus of the vehicle (e.g., headlights and windshield wipers).

Recent studies on vision-based driver assistance systems [3–10] attempt to identify vehicles, obstacles, traffic signs, raindrops, pedestrians, and other patterns in on-road traffic scenes from captured image sequences using image processing and pattern recognition techniques. By adopting different concepts and definitions of interesting objects on the road, applying different techniques to capture image sequences can detect vehicles or obstacles. In previous studies on detecting vehicles in an image sequence for driving safety, the vehicle detection process is mostly conducted by searching for specific patterns in the images based on typical features of vehicles, such as edge features, shape templates, symmetrization, or vehicles’ surrounding bounding boxes [3–10]. However, most studies on this topic have focused on detecting vehicles under daytime road environments.

At night, and under darkly illuminated conditions in general, headlights and taillights are often the only salient features of moving vehicles. In addition, many other sources of illumination that coexist with vehicle lights are found in road environments, including street lamps, traffic lights, and road reflector plates. These non-vehicle illumination objects make it difficult to obtain accurate cues for detecting vehicles in nighttime road scenes. To detect salient objects in nighttime traffic surveillance applications, Beymer *et al*. [11] applied a feature-based technique that extracts and tracks the corner features of moving vehicles instead of their entire regions. This approach works in both daytime and nighttime traffic environments, and is more robust to partial or complete occlusions. However, it suffers from high computational costs because it must simultaneously process numerous features of moving vehicles. Huang *et al*. [12] used block-based contrast analysis and inter-frame change information to detect salient objects at night. This contrast-based method can effectively detect outdoor objects in a surveillance area using a stationary camera. However, contrast and inter-frame change information are sensitive to the lighting effects of moving vehicle headlights, which often results in erroneous vehicle detection.

Recently, vehicle lights have been used as salient features for nighttime vehicle detection applications for driver assistance systems [13–15]. Stam *et al.* [13] developed an optical sensor array based system for detecting the vehicle lights. Based on an optical sensor array system set up on the vehicle, lighting objects appearing in the viewable area ahead of the host vehicle are imaged on the sensor array. Then a set of pre-determined thresholds are utilized to label bright-spotted pixels having gray intensities above the thresholds to determine the appearance of target vehicles. In Eichner and Breckon’s headlight detection method [14], a rule-based lighting object detection approach is conducted by segmenting the lighting objects using a fixed threshold along with a headlight pairing rules, and then a temporal tracking is combined to refine the detection results. The above-mentioned techniques can detect the appearance of vehicles under a nighttime road environment with few lighting sources. However, because these techniques use a set of fixed threshold values which were configured beforehand, they are unable to adaptively adjust the selection of threshold values to match different nighttime lighting conditions. Therefore, their reliability in handling the circumstances where road environments have various lighting conditions, is limited. To improve the feasibility on nighttime vehicle light detection, O’Malley *et al.* [15] presented a taillight detection approach, which adopts the hue-saturation-value (HSV) color features for taillight detection and integrates a Kalman filter tracking process. By integrating the HSV color features, this approach can efficient detect taillights with red lighting characteristics under various road environments, including urban, rural, and motorway environments. However, the utilization of multiple color components of HSV color spaces requires additional computational costs on color transformation because of the floating-point computation of the HSV color features, and the Kalman filtering also suffers large computational costs on numerical and inverse matrix computations. Thus, this approach is inappropriate for implementing on the portable embedded systems with limited computational resources.

Most of the previous systems are implemented on personal computer (PC)-based platforms, and therefore lack the portability and flexibility required for installation in vehicular environments. The computational power and flexibility of embedded systems have recently increased significantly because of the development of system-on-chip technologies, and the advanced computing power of newly released multimedia devices. Thus, performing real-time vision-based vehicle and object detection in driver safety applications has become feasible on modern embedded platforms for driver assistance systems [16–18].

In current requirements for driver assistance systems, driving event data recording is also an important functionality for traffic accident reconstruction. Unfortunately, most previously developed systems do not integrate the driving event recording functions with the driver assistance functions into a stand-alone portable system. Efficiently recording the real-time image sequences of traffic conditions with driver actions is essential to meeting the demands of monitoring and recording driving event data. For this purpose, the transform coding technique is the most popular method for compressing monitoring image frames. As the fundamental development of this field, discrete cosine transform (DCT)-based coding has been commonly used, and has since become an element of the JPEG image compression standard. Accordingly, it has been applied to numerous electronic devices today. Researchers have recently demonstrated that discrete wavelet transform (DWT)-based transform coding outperforms DCT-based methods [19–28]. Hence, newly developed image coding methods, such as the still image compression standard JPEG2000 [25,26] and the video coding method standard MPEG-4 [27,28], adopt concepts based on DWT features. The lifting scheme [23] nearly halves the time required to perform DWT computations. Therefore, this scheme has been incorporated into DWT-based image compression techniques. For example, zero-tree coding methods [21–24] can achieve the most coding efficiency to both DWT and DCT-based transform coding techniques.

However, traffic event video recording based on these image and video coding techniques, in addition to vision-based function modules, on a portable embedded system still suffers from computational problems. This is primarily because of the limited computational resources of embedded platforms. Recording traffic event videos during the long driving times requires huge storage space, and the storage space on an embedded portable platform is limited and expensive. Based on the requirements of traffic accident reconstruction, the system must record critical event videos of potential accidents. Thus, the limited storage space on a portable embedded system can be used efficiently, and the responsibility of possible traffic accidents can be more effectively reconstructed and identified. In this manner, the proposed vision-based nighttime vehicle detection and driver warning approaches can enable the accurate and timely determination of possible traffic accidents. Therefore, the proposed system can efficiently activate the traffic event video recording process when possible traffic accidents might occur as a result of driver negligence or inappropriate driving behaviors. To provide a satisfactory solution for these issues, this study adopts a heterogamous dual-processor embedded system platform, and the traffic event video recording function and vision-based driver assistance modules, including vehicle detection, traffic condition analysis, and driver warning functions are implemented and optimized using the computational resources of the two heterogamous processors.

This study proposes a vision-based intelligent nighttime driver assistance and surveillance (VIDASS) system. The proposed VIDASS system includes the computer vision and sensing techniques of nighttime vehicle detection, collision warning determination, and traffic event recording functions by processing the road-scene frames in front of the host car, which are captured from the CCD sensors mounted inside the host vehicle. These proposed vision-based sensing and processing technologies are implemented as a set of embedded software component and modules based on a component-based system framework, and are integrated and performed on an ARM-DSP heterogamous dual-core embedded platform. Peripheral devices, including image grabbing devices, network communication modules, and other in-vehicle control devices, are also integrated to produce an in-vehicle embedded vision-based nighttime driver assistance and surveillance system. Accordingly, the goals and features of the proposed embedded driver assistance system are given as follows:

1. Effective detection and analysis road environment based on image segmentation, object recognition, and motion analysis;
2. Real-time event recording using efficient video compression and storage technology;
3. Configurable software framework to achieve the extension of the convenience and scalability;
4. A low-cost and high performance night driving assistance system implemented on a heterogamous dual-processor (ARM-DSP core) embedded system platform.

Experimental results show that the proposed system provides both efficiency and feasible advantages for integrated vehicle detection, collision warning, and traffic event recording for driver surveillance in various nighttime road environments and different traffic conditions.

## 2. The Proposed Nighttime Driver Assistance and Event Recording System

**Figure 1.**
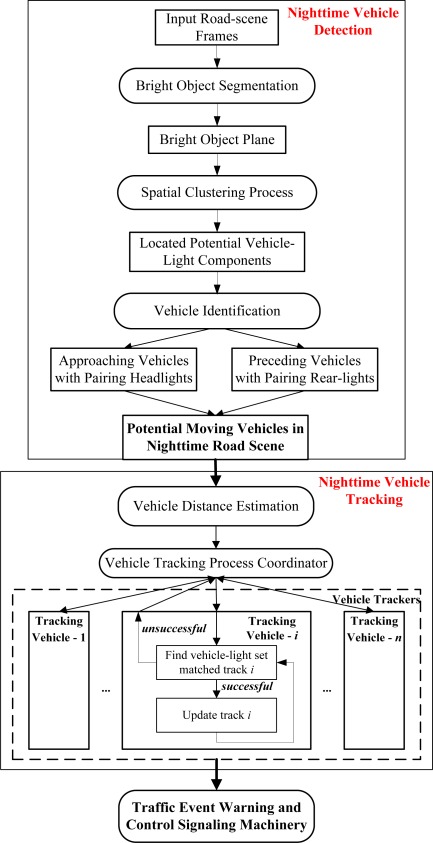
System block diagram.

The proposed vision-based intelligent nighttime driver assistance and surveillance (VIDASS) system integrates effective vision-based sensing and processing modules, including nighttime vehicle detection, collision warning determination, and event recording functionalities. These functions are implemented to identify target vehicles in front of the host car, estimating their distances, determine possible collision accidents, and record traffic event videos. The real-time vision-based sensing and processing modules of the proposed VIDASS system include bright object segmentation, spatial clustering process, rule-based vehicle identification, vehicle distance estimation, and traffic event warning and control signaling machinery. The following subsections describe these features. Figure 1 shows a flow diagram of the proposed vision processing modules for the VIDASS system.

### 2.1. Bright Object Segmentation Module

**Figure 2.**
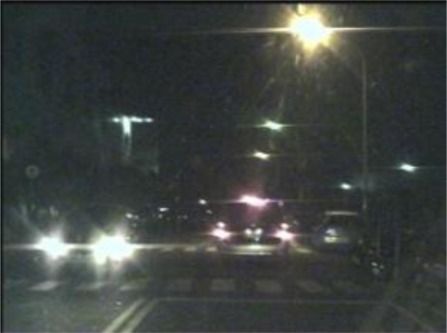
A sample nighttime road scene.

The input image sequences are captured from the vision system. These sensed frames reflect nighttime road environments appearing in front of the host car. Figure 2 shows a sample nighttime road scene taken from the vision system. In this sample scene, two vehicles are on the road. The left vehicle is approaching in the opposite direction on the neighboring lane, and the right vehicle is moving in the same direction as the camera-assisted host car.

**Figure 3.**
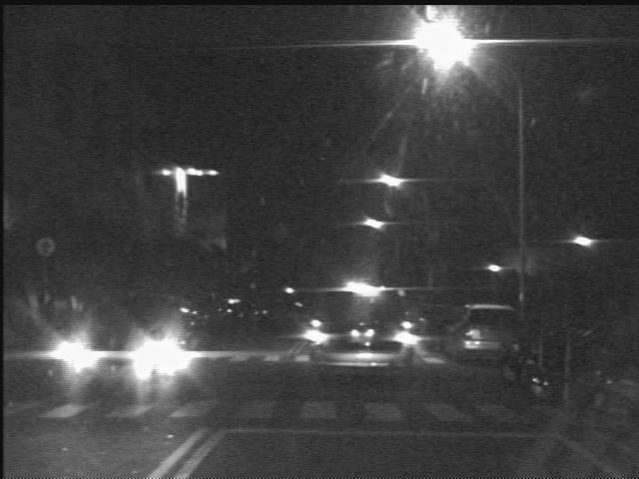
A sample grayscale nighttime road scene.

The task of the bright object segmentation module is to extract bright objects from the road scene image to facilitate subsequent rule-based analysis. To reduce the computation cost of extracting bright objects, the module first extracts a grayscale image (Figure 3) (*i.e*., the Y-channel) of the captured image by performing a RGB-to-Y transformation.

To extract these bright objects from a given transformed gray-intensity image, pixels of bright objects must be separated from other object pixels of different illuminations. Thus, an effective multilevel thresholding technique is required to automatically determine the appropriate number of thresholds for segmenting bright object regions from the road-scene image. For this purpose, we have proposed an effective automatic multilevel thresholding technique for fast region segmentation [29]. This technique can automatically decompose a captured road-scene image into a set of homogeneous thresholded images based on an optimal discriminant analysis concept. Extensive studies based on this optimal discriminant analysis concept have also been efficiently used in various object segmentation and analysis applications, such as text extraction for document image analysis [30], biofilm image segmentation [31,32], and multi-touch sensing detection applications [33].

**Figure 4.**
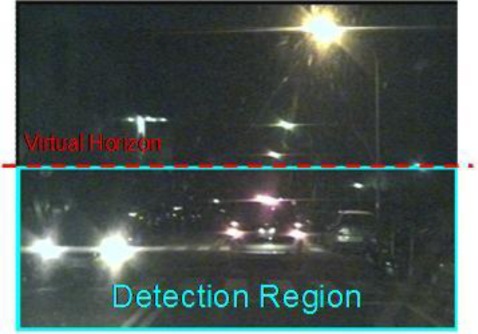
The detection region and virtual horizon for bright object extraction in Figure 2.

**Figure 5.**
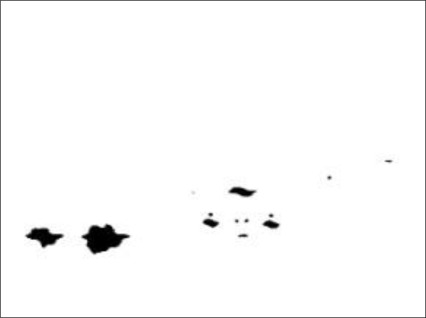
Results of performing the bright object segmentation module on the road scene images in Figure 2.

Accordingly, to screen out non-vehicle illuminant objects such as street lamps and traffic lights located above half of the vertical y-axis (*i.e.*, the “horizon”), and save the computation cost, the bright object extraction process is only performed on the bright components located under the virtual horizon (Figure 4). Accordingly, as Figure 5 shows, after applying the bright object segmentation module on the sample image in Figure 2, pixels of bright objects are successfully separated into thresholded object planes under real illumination conditions.

### 2.2. Spatial Analysis and Clustering Process Module

To identify potential vehicle-light components after performing bright object segmentation, a connected component extraction process is then performed on the bright object plane to locate the connected-components [34] of the bright objects. This process attempts to identify the horizontal-aligned vehicle lights; hence, a spatial clustering process is applied to the connected-components to cluster them into several meaningful groups. The resulting group includes a set of connected components, and may consist of vehicle lights, traffic lights, road signs, and other illuminated objects that frequently appear in nighttime road scenes. These groups are then processed by the vehicle light identification process to identify the actual moving vehicles. The following steps outline the proposed projection-based spatial clustering process:

1. *C_i_* denotes one certain bright connected-component to be processed.
2. *CG_k_* denotes a group of bright components, *CG_k_* = {*C_i_*, *i* = 0, 1, 2,…, *p*}, and the total number of connected components contained in *CG_k_* is denoted as *N_cc_*(*CG_k_*) .
3. The locations of the bounding boxes of a certain component *C_i_* employed in the spatial clustering process are the top, bottom, left and right coordinates of these components, and these locations are denoted as *t*(*C_i_*), *b*(*C_i_*), *l*(*C_i_*), and *r*(*C_i_*), respectively.
4. The width and height of a bright component *C_i_* are denoted as *W*(*C_i_*) and *H*(*C_i_*), respectively.
5. The horizontal distance *D_h_* and vertical distance *D_v_* between two bright components are defined as:
  (1) $$D_{h}(C_{i},C_{j})=\text{max}\left[l(C_{i}),l(C_{j})\right]-\text{min}\left[r(C_{i}),r(C_{j})\right]$$
  (2) $$D_{v}(C_{i},C_{j})=\text{max}\left[t(C_{i}),t(C_{j})\right]-\text{min}\left[b(C_{i}),b(C_{j})\right]$$
  If the two bright components are overlapping in the horizontal or vertical direction, then the value of *D_h_*(*C_i_*,*C_j_*) or *D_v_*(*C_i_*,*C_j_*) is negative.
6. The degree of overlap between the vertical projections of the two bright components can be computed as:
  (3) $$P_{v}(C_{i},C_{j})=-D_{v}(C_{i},C_{j})/\text{min}\left[H(C_{i}),H(C_{j})\right]$$

To preliminarily screen out non-vehicle illuminant objects such as street lamps and traffic lights, first filter out the bright components located above one-third of the vertical y-axis (*i.e.*, only the bright components located under the constraint line), Thus, only the “virtual horizon” in Figure 4 is considered. This is because vehicles located at a distant place on the road become very small light “points”, and “converge” into a virtual horizon.

To determine the moving directions of detected vehicles, it is necessary to identify the bright components at potential headlights and taillights before performing the respective analyses. The distinguishable characteristics of taillights are red illuminated lights. However, when the preceding vehicles are close to the camera-assisted car (*i.e*., within 30 m), their taillights cause “blooming effects” in CCD cameras, and are usually too bright to appear as white objects in the captured images. As a result, only pixels located around the components of potential taillights have a distinguishable red appearance. Hence, the following *red-light criterion* is used to check if a bright component contains a potential taillight object. This criterion is determined as:

(4) $$R_{p}(C_{i})-T_{\mathrm{red}}\;\;\;>\;\;\;\text{both}\;\;\;G_{p}(C_{i})\;\;\;\text{and}\;\;\;B_{p}(C_{i})$$

where *T_red_* is a predetermined threshold, and *R_p_*(*C_i_*), *G_p_*(*C_i_*), and *B_p_*(*C_i_*) respectively represent the average intensities of the *R*, *G*, and *B* color frames of the pixels located at the peripheral of a given bright component *C_i_*. The value of *T_red_* is chosen as 10 to discriminate potential taillights and other bright components. If a bright component *C_i_* satisfies the red-light criterion, then *C_i_* is tagged as a *red-light* component; otherwise, it is tagged as a *non-red-light* component.

The connected components of bright objects are then recursively merged and clustered into bright component groups *CGs* if they have the same light tags, are horizontally close to each other, vertically overlapped, and aligned. In other words, if neighboring bright components satisfy the following conditions, they are merged with each other and clustered as the same group *CG*:

1. They have the same tags (*i.e*., both are either *red-light* or *non-red-light* components).
2. They are horizontally proximate:
  (5) $$D_{h}(C_{i},C_{j})\;\;\;<\;\;\;T_{d}\times \text{max}\left(H(C_{i}),H(C_{j})\right)$$
3. They are highly overlapped in vertical projection profiles:
  (6) $$P_{v}(C_{i},C_{j})\;\;\;>\;\;\;T_{p}$$
4. They have similar heights:
  (7) $$H(C_{S})/H(C_{L})\;\;>\;\;T_{h}$$

where *C_S_* is has a smaller height among the two bright components *C_i_* and *C_j_*, whereas *C_L_* has a taller height.

**Figure 6.**
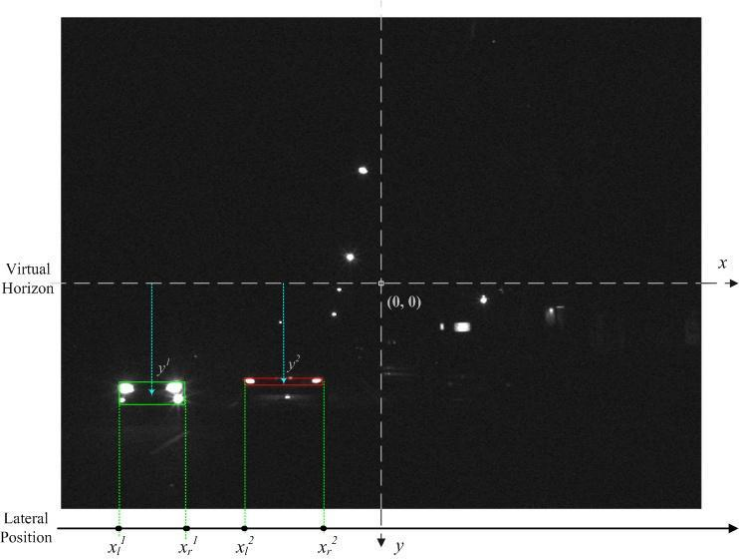
The spatial clustering process of the bright components of interest.

In this study, *T_d_*, *T_p_*, and *T_h_* represent the predetermined thresholds to determine the pairing characteristics of vehicle lights. These values are reasonably chosen as 3.0, 0.8, and 0.7, respectively. Figure 6 illustrates the processing results of the spatial clustering module applied to bright components. The spatial clustering process yields several groups of bright components, denoted as candidate vehicle-light groups.

### 2.3. Vehicle Tracking and Identification Module

These methods obtain the light groups of potential vehicles in each captured frame. However, because sufficient features of some potential vehicles may not be immediately obtained from single image frames, a tracking procedure must be applied to analyze the information of potential vehicles from more successive image frames. The tracking information can then be used to refine the detection results and suppress occlusions caused by noise and errors during the bright object segmentation process and spatial clustering process. This tracking information can also be applied to determine useful information such as the direction of movement, positions, and relative velocities of potential vehicles entering the surveillance area.

The proposed vehicle tracking and identification module in the proposed vision system includes two phases. First, the phase of potential vehicle tracking associates the motion relation of potential vehicles in succeeding frames by analyzing their spatial and temporal features. The phase of vehicle recognition subsequently identifies actual vehicles among the tracked potential vehicles.

#### 2.3.1. Potential Vehicle Tracking Phase

Considering only the detected object regions of potential vehicles in single frames may lead to the following problems in the segmentation process and the spatial clustering process: (1) a preceding vehicle may be too close to another vehicle moving parallel or street lamps, so that they may be occluded during the segmentation process, and thus, detected as one connected region; (2) an oncoming vehicle may be passing so close to the host car that it may be occluded by the reflected beams on the road, and hence be merged into one large connected region; and (3) the headlight set or taillight set of a vehicle may include multiple light pairs, and they may not be immediately merged into a single group by the spatial clustering process.

The motion of object regions of potential vehicles makes it possible to progressively process and refine the detection results of the potential vehicles by associating them in sequential frames. Therefore, this study presents a tracking process for potential vehicles that can effectively handle these problems. The tracking information of potential vehicles is also provided to the following vehicle identification and motion analysis processes to determine appropriate relative motion information regarding the target vehicles ahead of the host car.

When a potential vehicle is first detected in the field of view in front of the host car, a tracker is created to associate this potential vehicle with those in subsequent frames by applying spatial-temporal features. The following points describe the features used in this tracking process:

- $P_{i}^{t}$ denotes the *i^th^* potential vehicle appearing in front of the host car in frame *t*, and the bounding box, which encloses all the bright components contained in $P_{i}^{t}$, is denoted as $B(P_{i}^{t})$.
- The location of $P_{i}^{t}$ employed in the tracking process is represented by its central position, and can be expressed as:
  (8) $$P_{i}^{t}=\left(\frac{l\left(B(P_{i}^{t})\right)+r\left(B(P_{i}^{t})\right)}{2},\frac{t\left(B(P_{i}^{t})\right)+b\left(B(P_{i}^{t})\right)}{2}\right)$$
- The overlapping score of the two potential vehicles $P_{i}^{t}$ and $P_{j}^{t^{\prime }}$, detected at two different times *t* and *t’*, respectively, can be computed based on their area of intersection:
  (9) $$S_{o}(P_{i}^{t},P_{j}^{t^{\prime }})=\frac{A\left(P_{i}^{t}\cap P_{j}^{t^{\prime }}\right)}{\mathrm{Max}\left(A(P_{i}^{t}),A(P_{j}^{t^{\prime }})\right)}$$
- The size-ratio feature of the enclosing bounding box of $P_{i}^{t}$ is defined as:
  (10) Rs(Pit)=W(B(Pit))H(B(Pit))
- The symmetry score of the two potential vehicles $P_{i}^{t}$ and $P_{j}^{t^{\prime }}$ can then be obtained by:
  (11) Ss(Pit,Pjt′)=Rs(PS)Rs(PL)
  where *P_S_* is the one with the smaller size among the two potential vehicles $P_{i}^{t}$ and $P_{j}^{t^{\prime }}$, and *P_L_* is the larger one.

In each iteration of the tracking process for a newly incoming *frame t*, the potential vehicles appearing in the incoming frame, denoted as $P^{t}=\left\{P_{i}^{t}|i=1,\ldots ,k^{\prime }\right\}$, are analyzed and associated with the set of potential vehicles that have already been tracked in the previous frame *t* − 1, denoted as ${\mathrm{TP}}^{t-1}=\left\{{\mathrm{TP}}_{j}^{t-1}|j=1,\ldots ,k\right\}$. The set of the tracked potential vehicles **TP***^t^* is subsequently updated according to the following process.

During the tracking process, a tracked potential vehicle might be in one of five possible states. The vehicle tracking process then applies different operations according to the given states of each tracked potential vehicle in each frame. The tracking states and associated operations for the tracked potential vehicles are as follows:

- **Update**: When a potential vehicle $P_{i}^{t}\in P^{t}$ in the current frame can match a tracked potential vehicle ${\mathrm{TP}}_{j}^{t-1}\in {\mathrm{TP}}^{t-1}$, then the tracker updates the set of the tracked potential vehicles **TP***^t^* by associating $P_{i}^{t}$ with the tracker ${\mathrm{TP}}_{j}^{t}$ if the following matching condition is satisfied. The *matching condition* is defined as:
  (12) $$m\left(P_{i}^{t},{\mathrm{TP}}_{j}^{t-1}\right)\;\;>\;\;0.6$$
  where the matching score $m\left(P_{i}^{t},{\mathrm{TP}}_{j}^{t-1}\right)$ is computed as:
  (13) $$m\left(P_{i}^{t},TP_{j}^{t-1}\right)=w_{o}\cdot S_{o}(P_{i}^{t},TP_{j}^{t-1})+w_{s}\cdot S_{s}(P_{i}^{t},{\mathrm{TP}}_{j}^{t-1})$$
  where *w_o_* and *w_s_* represent the weights of the overlapping score and the size-ratio feature, and are set at 0.5 and 0.5, respectively.
- **Appear**: If a newly identified potential vehicle $P_{i}^{t}\in P^{t}$ cannot match any ${\mathrm{TP}}_{j}^{t-1}\in {\mathrm{TP}}^{t-1}$ in the previous time, then a new tracker for this potential vehicle is created and appended to the updated set **TP***^t^*.
- **Merge**: A potential vehicle $P_{i}^{t}$ at the current frame matches multiple tracked regions of potential vehicles ${\mathrm{TP}}_{j}^{t-1}$, ${\mathrm{TP}}_{j+1}^{t-1}$…, ${\mathrm{TP}}_{j+n}^{t-1}$. This situation may occur if the headlight set or taillight set of a single vehicle consists of multiple light pairs that were not clustered into a single group in the previous frame, but are correctly merged by the spatial clustering process in the current frame. Therefore, if ${\mathrm{TP}}_{j}^{t-1}$, ${\mathrm{TP}}_{j+1}^{t-1}$…, and ${\mathrm{TP}}_{j+n}^{t-1}$ all satisfy the *matching condition* in Equation (12) with $P_{i}^{t}$, then they are merged into a single tracker ${\mathrm{TP}}_{i}^{t}$, and the tracker set **TP***^t^* is also updated.
- **Split**: One tracked potential vehicle ${\mathrm{TP}}_{j}^{t-1}$ in the previous frame is split into multiple regions of potential vehicles $P_{i}^{t}$, $P_{i+1}^{t}$ …, $P_{i+m}^{t}$ in the current frame. This situation may occur if a vehicle is moving too close to other parallel moving vehicles, street lamps, or reflected beams on the road so that they are occluded, but are later correctly split by the segmentation process. Thus, the matching condition between each ${\mathrm{TP}}_{j}^{t-1}\in {\mathrm{TP}}^{t-1}$ can be evaluated with $P_{i}^{t}$, $P_{i+1}^{t}$…, $P_{i+m}^{t}$. If any one of these regions of potential vehicles matches ${\mathrm{TP}}_{j}^{t-1}$, then that potential vehicle is associated with ${\mathrm{TP}}_{j}^{t}$, and other non-matched potential vehicles begin their new trackers ${\mathrm{TP}}_{j}^{t}$, ${\mathrm{TP}}_{j+1}^{t}$…, ${\mathrm{TP}}_{j+m-1}^{t}$ in the updated tracker set **TP***^t^*.
- **Disappear**: A existing tracker of potential vehicle ${\mathrm{TP}}_{j}^{t-1}\in {\mathrm{TP}}^{t-1}$ cannot be matched by any newly coming potential vehicles $P_{i}^{t}\in P^{t}$. A tracked potential vehicle may be temporarily occluded in some frames, but soon reappear in subsequent frames. Thus, to prevent such a potential vehicle from being regarded as a newly appearing potential vehicle, its tracker is retained in the subsequent three frames. If a tracker of potential vehicle ${\mathrm{TP}}_{j}^{t-1}$ fails to match any potential vehicles $P_{i}^{t}\in P^{t}$ for more than three succeeding frames, then this potential vehicle is judged to have disappeared and its tracker is removed from the tracker set **TP***^t^* in the following frames.

#### 2.3.2. Vehicle Identification from Tracking Phase

To distinguish actual vehicles in each frame, a rule-based recognition process is applied to each of the potential tracked vehicles to determine whether it comprises actual vehicle lights or other illuminated objects. If a tracked potential vehicle *TP_j_* contains a set of actual vehicle lights that reveal an actual vehicle, then the following discriminating rules of statistical features must be satisfied:

- Because an on-road vehicle can be approximately modeled as a rectangular patch, the enclosing bounding box of the potential vehicle must form a horizontal rectangular shape. In other words, the size-ratio feature of the enclosing bounding box of *TP_j_* must satisfy the following condition:
  (14) $$\tau_{r1}\leq W\left({\mathrm{TP}}_{j}\right)/H\left({\mathrm{TP}}_{j}\right)\leq \tau_{r2}$$
  where the threshold τ*_r_*_1_ and τ*_r_*_2_ on the size-ratio condition are selected as 2.0 and 10.0 to identify the rectangular-shaped appearance of paired vehicle lights.
- The number of lighting components in *TP_j_* should be symmetrical and well-aligned. Thus, the number of these components should be in reasonable proportion to the size of the size-ratio feature of its enclosing bounding box, and the following alignment condition must be satisfied:
  (15) $$\tau_{a1}\left(\frac{W({\mathrm{TP}}_{j})}{H({\mathrm{TP}}_{j})}\right)\leq N_{\mathrm{cc}}\left({\mathrm{TP}}_{j}\right)\leq \tau_{a2}\left(\frac{W({\mathrm{TP}}_{j})}{H({\mathrm{TP}}_{j})}\right)$$
  where the thresholds τ_*a*1_ and τ_*a*2_ are set at 0.4 and 2.0, respectively, based on an analysis of the typical visual characteristics of most vehicles during nighttime driving.
- Oncoming vehicles usually appear on the left side of the road; thus, *TP_j_* containing a non-red-light pair at the right side of the red-light pairs should be ignored. Hence, the headlight-pair locating condition is defined as:
  (16) $$l_{\mathrm{head}}\left({\mathrm{TP}}_{j}\right)<l_{\mathrm{tail}}\left({\mathrm{TP}}_{j}\right)$$
  where *l*(*TP_j_*) is denoted as a left coordinate.

These discriminating rules were obtained by analyzing many experimental videos of real nighttime road environments in which vehicle lights appear in different shapes, sizes, directions, and distances. The values of the thresholds used for these discriminating rules were determined to yield good performance in most nighttime road environments.

Accordingly, the proposed system recognizes a tracked potential vehicle as an actual vehicle when these vehicle identification rules are satisfied. A tracked vehicle is no longer recognized as an actual vehicle when it cannot satisfy the vehicle identification criteria over a number of frames (three frames in general) or when it disappears from the field of view.

### 2.4. Vehicle Distance Estimation Module

For estimating the distance between the camera-assisted car and detected vehicles, the proposed module applies the perspective range estimation model of the CCD camera introduced in [35]. The origin of the virtual vehicle coordinate system appears at the central point of the camera lens. The *X* and *Y*-coordinate axes of the virtual vehicle coordinate are parallel to the *x* and *y*-coordinates of the captured images, and the *Z*-axis is placed along the optical axis and perpendicular to the plane formed by the *X* and *Y* axes. A target vehicle on the road at a distance *Z* in front of the host car projects to the image at the vertical coordinate *y*. Thus, the perspective single-camera range estimation model presented in [32] can be used to estimate the *Z*-distance in meters between the camera-assisted car and vehicle detected using the equation:

(17) Z=k⋅((f⋅H)/y)

where *k* is a given factor for converting from pixels to millimeters for the CCD camera mounted on the car at the height *H*, and *f* is focal length in meters.

### 2.5. Event-Driven Traffic Data Recording Subsystem

**Figure 7.**
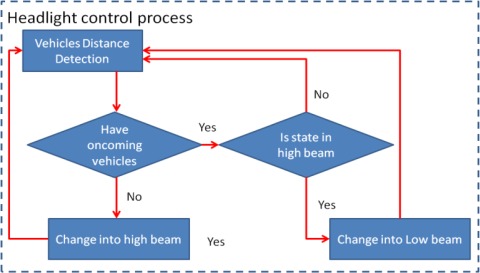
Headlight control process work flow.

**Figure 8.**
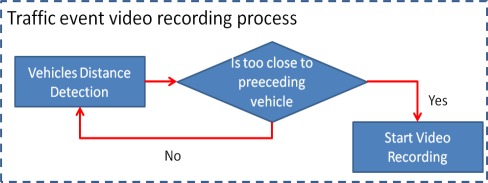
Traffic event video recording process work flow.

The proposed traffic event warning and recording subsystem is an automatic control process in the proposed system. This automatic control process includes a vehicle headlight control process and a traffic event video recording process. When any oncoming vehicles are detected, the headlight control process automatically switches the headlights to low beam, and then reverts to high beams once the detected vehicles leave the detection zone. The warning voice and traffic event video recording process are also activated to notify drivers to slowly decelerate when the distance from the detected preceding vehicles is too small, and to record a traffic event video using the MPEG4 video codec [27,28]. Figures 7 and 8 present flowcharts of the headlight control process and the traffic event video recording process, respectively.

## 3. Embedded System Implementations

This section describes the implementation of the proposed VIDASS system. Subsection 3.1 presents the core architecture of the VIDASS system, including the integration of t with an heterogamous dual-core embedded platform with an embedded Linux OS and Qt graphical user interface (GUI) for user-friendly control machinery. This study also applies a component-based framework to implement the software framework of the proposed system. Subsection 3.2 describes the software architecture and implementation of the proposed VIDASS system.

### 3.1. Core Architecture

The proposed VIDASS system includes a set of vision-based modules and subsystems as described in the previous section, and these vision modules require an embedded portable platform that can provide efficient and optimized video-intensive computational resources and feasible embedded software development tools. For the aspects of hardware platform selection, the ARM general processors are famous for their low cost, low power consumption, extensive general-purpose IO control facilities, and sufficient OS awareness. However, the hardware architecture of ARM processors is designed to support general-purpose applications, and is therefore unsuitable for multimedia applications, such as image and video processing applications that cost large numerical computations on a large amount of multimedia data. In contrast, a digital signal processor (DSP), such as Texas Instruments (TI) C64x+ series, can provide optimized and accelerated computational instructions and units for video-intensive algorithms and multimedia data processing applications, although it lacks sufficient OS awareness compared with ARM processors. Thus, a complementary combination of the ARM general processor and the digital signal processor can provide a best-fitted solution for developing a vision-based system with the embedded OS, GUI module, and peripheral IO control facilities. In this way, we can divide the VIDASS system into a set of execution modules on the ARM and DSP processors according to their functional and computational properties to obtain effective performance and economic hardware costs.

**Figure 9.**
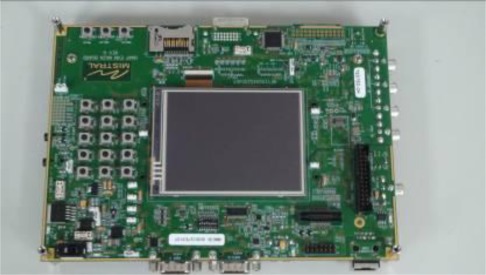
The TI OMAP3530 Embedded Experimental Platform.

In this study, the proposed VIDASS system was implemented on an ARM-DSP heterogamous dual-core embedded platform, the TI OMAP3530 platform (Figure 9) [36]. The TI OMAP3530 platform is an efficient solution for portable and handheld systems from the TI DaVinci™ Digital Video Processor family. This platform consists of one Cortex A8 ARM-based general-purpose processor with a 600 MHz operational speed and one TI C64x+ digital signal processor with a 430 MHz operational speed on a single chip. This platform also includes 256 MB of flash ROM memory for storing the embedded Linux OS kernel, Qt-based GUI system, and the proposed vision-based software modules, and 256 MB of DDR memory for executing the software modules. In addition to the main platform, peripheral devices such as image grabbing devices, LCD touchscreen panel, mobile communication module, and other in-vehicle control devices are also integrated to accomplish an in-vehicle embedded vision-based nighttime driver assistance and surveillance system.

As for the implementation issue on the embedded platform with heterogamous processors, the processor architectures, instruction sets, and clock rates of the ARM and the DSP are different. Thus, the efficient system-level integration of these two heterogamous processors on multimedia application software remains a problem for technicians developing efficient solutions. From a developer’s viewpoint, it is necessary to develop the main components of the system (such as the main application, GUI, and peripheral IO control facilities) on the ARM-side, in addition to the embedded operating system (OS) environments (such as the embedded Linux OS). Thus, it would be better to adopt the digital signal processor as a hardware computing resource in the embedded OS at the ARM-side, and assign the computational jobs of the desired vision-based algorithms and event video recording functions to be performed on the DSP-side. Transferring the multimedia data between the ARM and DSP can be achieved using the shared memory machinery and direct memory accesses. Based on these concepts, the TI provides a series of Linux Digital Video Software Development Kits (DVSDK) solutions for developing multimedia intensive application systems on ARM-DSP heterogamous dual-core platforms [37] of TI DaVinci™ Digital Video Processor family. The software architecture diagram provide in [37] shows the core architecture of the DVSDK. Thus, the proposed VIDASS system was implemented on a TI OMAP3530 platform with the DVSDK architecture. Based on this software development architecture, the proposed VIDASS system can be easily migrated, customized and extended on various embedded platforms of TI DaVinci™ Digital Video Processor family by the system developers for different demands of nighttime driver assistance applications.

### 3.2. Software Implementation

**Figure 10.**
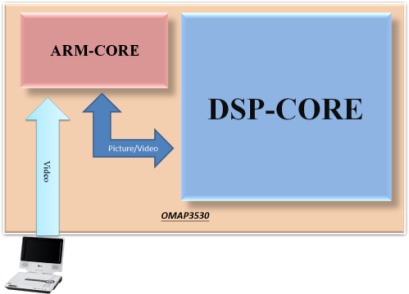
The software architecture of the VIDASS system on the ARM-DSP heterogamous dual-core embedded platform.

For the embedded software implementation on the OMAP3530 platform, the vision-based subsystem modules of the proposed VIDASS system (as described in the previous section) are implemented on the DSP-side to guarantee the real-time computational performance of image and vision processing modules. The main GUI system and peripheral IO control facilities were executed on the ARM-side along, in addition to the embedded Linux. The proposed VIDASS system uses the TI DVSDK software architecture to accomplish the real-time vision-based computing modules on the ARM-DSP heterogamous dual-core embedded platform, as Figure 10 illustrates. The study adopts a component-based framework to develop the embedded software framework of the proposed VIDASS system. The VIDASS software framework includes the following embedded vision computing modules: the bright object segmentation (BOS) module, the spatial analysis and clustering process (SCP) module, the vehicle tracking and identification (VTI) module, the vehicle distance estimation (VDE) module, and the event-driven traffic data recording subsystem (ETDS). The advantages of using a component-based system include the ease of updating new function modules and extending the system to any subsequent developments of new vision-based assistance functional modules.

**Figure 11.**
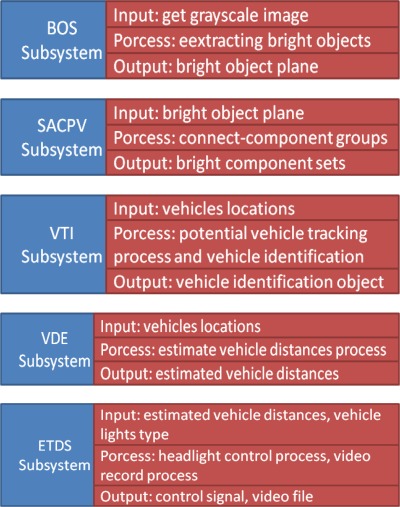
Software modules of the proposed VIDASS system.

Figure 11 shows the software modules of the proposed VIDASS system. This figure shows that, based on the software modules, the proposed VIDASS system includes five processing stages: In the first stage, the BOS module obtains the grayscale image sequences captured from the vision system, and then applies the segmentation process to extract bright objects of interest.

The BOS module then exports the obtained bright object plane to the following stage. In the second stage, the SACPV module receives the bright object plane from the BOS module, applies a connected-component extraction process to group the bright objects into meaningful sets, and then sends out the resulting bright component sets. The third stage is the VIL module, which obtains the bright component sets from SACPV module and performs the rule-based identification process to detect and verify the oncoming and preceding vehicles. The VIL module then sends the detected vehicles and their locations out to the VDE module. In the fourth stage, the VDE module determines the relative distances between the host car and the detected oncoming and preceding vehicles, and then provides the estimated vehicle distances to the following stage. The final stage is the ETDS module, which performs and activates the headlight control process and activates the traffic event video recording according using the MPEG4 codec implemented by the DSP codec library [36,37] based the information of detected vehicles and their related distances.

**Figure 12.**
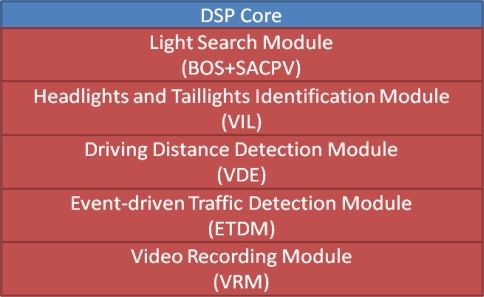
The software architecture of the vision computing modules on the DSP-core.

**Figure 13.**
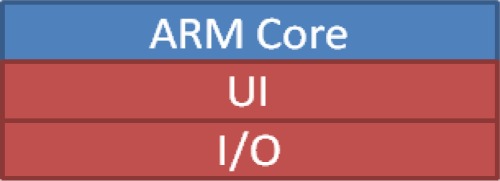
The software architecture of the main system on the ARM-core.

The experiments in this study implemented these vision computing modules on a DSP-core to guarantee the computational efficiency of the optimized video processing instruction libraries provided by TI. To set the vision computing modules as a set of executive computation entities on the DSP-core, these vision modules are realized as the “customized codecs” according to the TI CodecGengine standard [38]. Thus, these modules can serve as the computational entities and are efficiently executed on the DSP-core based on the DVSDK software architecture [37]. Therefore, these vision computing modules can be effectively run on the DSP-core (Figure 12). The main system software executed on the ARM-core (Figure 13) can also efficiently apply and control these modules to obtain the processing results of the traffic conditions in front of the host car.

**Figure 14.**
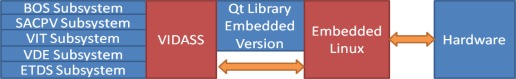
Proposed system software architecture.

The main system software on the ARM-core was performed and integrated on an embedded Linux OS kernel, the Qt GUI SDK library. To control the hardware peripherals, the proposed system activates the corresponding control messages to the device drivers on the embedded Linux kernel. The Linux kernel then signals the related hardware peripherals, such as GPIO signals, voice speakers, and communication devices. Thus, drivers can operate the proposed system using the touch panel and hardware peripherals. Figure 14 shows the overall software architecture of the proposed VIDASS system.

## 4. Experimental Results

**Figure 15.**
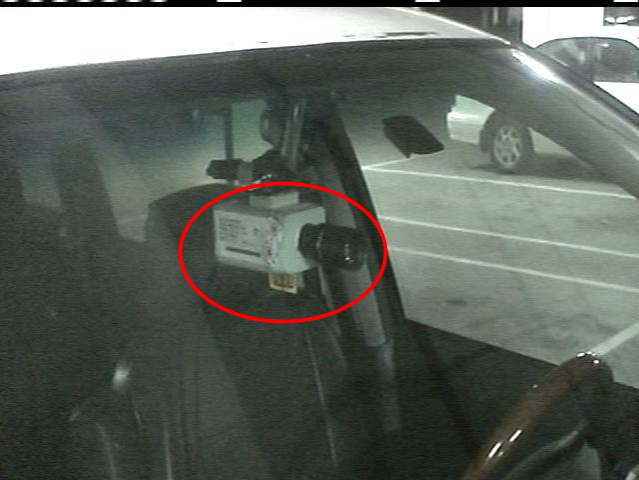
Proposed system software architecture.

The proposed vision-based techniques and modules of the VIDASS system were integrated and implemented on a TI IMAP3530 ARM-DSP dual-core embedded platform, which was set up on an experimental camera-assisted car. To make the proposed system operate well, the CCD camera for acquiring input image sequences of front road environments is mounted behind the windshield inside the experimental camera-assisted car with a sufficient height for capture a suitable region for covering the interesting driving lanes to be monitored, as shown in Figure 15. Then the view angle of the CCD camera is calibrated to be parallel to the ground for obtaining reliable features of target vehicles and the relative distances to them. In our experimental platform, the CCD camera is set at a 1.3 m height to the ground and parallel to the ground with an elevation angle of zero degree, while the focal length of the CCD camera is set as 10 mm. The peripheral devices, including image grabbing devices, mobile communication modules, and other in-vehicle control devices, were also integrated on the ARM-DSP dual-core embedded platform to accomplish an in-vehicle embedded vision-based nighttime driver assistance and surveillance system. The proposed vision system has a frame rate of 10 fps, and the size of each frame of captured image sequences is 720 pixels × 480 pixels per frame. The proposed system was tested on several videos of real nighttime road-scenes under various conditions.

**Figure 16.**
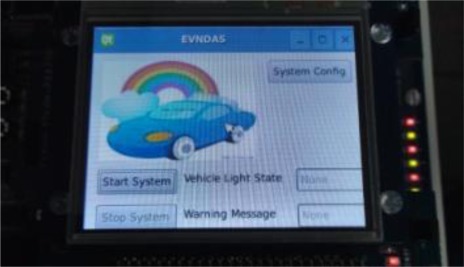
The user interface of the proposed VIDASS system.

Figure 16 shows the main system screen of the proposed VIDASS system, which consists of three buttons, including the functions of the system configuration, system starting, and system stopping. The system configuration sets system values such as the voice volume, control signaling, and traffic event video recording, whereas system starting and system stopping buttons start and stop the proposed system, respectively.

The experiments in this study analyzed nine nighttime traffic video clips captured in urban, rural, and highway road environments with various traffic flows and ambient illumination conditions (test videos 1–9 in Figures 17–25).

The types of ambient lighting conditions in the experimental videos include normal illumination conditions, bright illumination conditions, and dim illuminations. Experimental videos in the normal illumination conditions were captured in the normal lit road environments, where the lighting sources are streetlights and vehicle lights. Experimental videos in the bright illumination conditions were captured in the highly lit urban road environments, where the lighting sources comprise of large amount of streetlights, building lights, neon lights and vehicle lights; while dim illumination videos were captured in unlit highway or rural road environments with only a few streetlights and vehicle lights. Each video lasted approximately 5–10 min. The locations of the detected preceding vehicles in the experimental samples are indicated by red rectangles, whereas the oncoming vehicles are labeled as green rectangles. When the host car comes too close to the detected preceding vehicles, the ETDS process warns the driver to slow to avoid collision dangers, and activates the traffic event recording process. When the proposed system detects oncoming vehicles, the ETDS module automatically switches the headlights from high beam to low beam.

**Figure 17.**
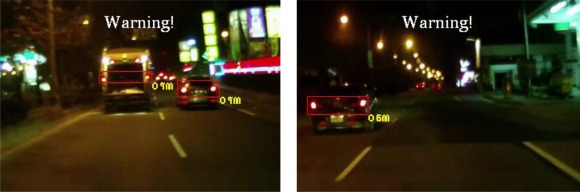
Results of vehicle detection and event determination for a nighttime rural road under a normal illumination with free-flowing traffic conditions (Test video 1).

**Figure 18.**
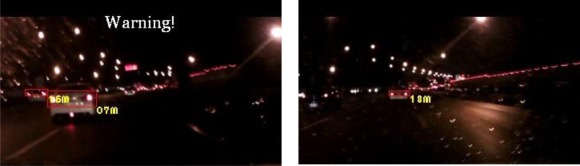
Results of vehicle detection and event determination for a nighttime rural road under a normal illumination with rainy and free-flowing traffic conditions (Test video 2).

**Figure 19.**
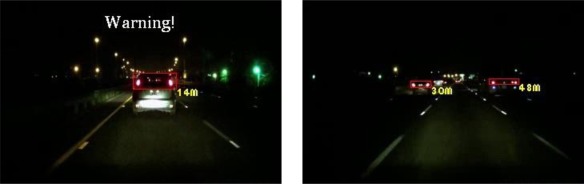
Results of vehicle detection and event determination for a nighttime rural road under a dim illumination with free-flowing traffic conditions (Test video 3).

First, the experimental videos in Figures 17–19 (Test videos 1–3) are evaluated at nighttime rural roads under normal and dim lighting conditions with free traffic flows. The snapshots in Figures 17–19 show that the proposed system correctly detects most of the preceding and oncoming vehicles, estimates their distances to the host car, and warns the driver to avoid possible collision dangers under different illumination conditions. The system also activates the event recording process when the host car drives too close to the target vehicles ahead.

**Figure 20.**
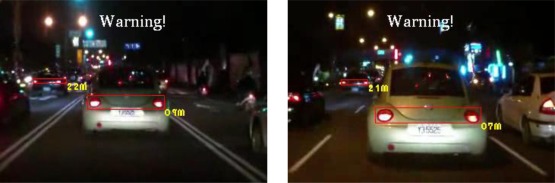
Results of vehicle detection and event determination for a nighttime urban road under normal illumination and congested traffic conditions (Test video 4).

**Figure 21.**
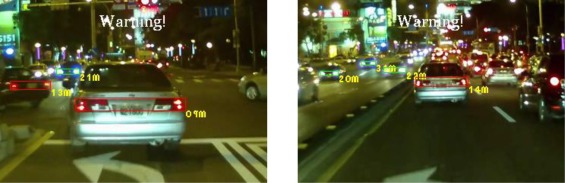
Results of vehicle detection and event determination for a nighttime urban road under bright illumination and congested traffic conditions (Test video 5).

**Figure 22.**
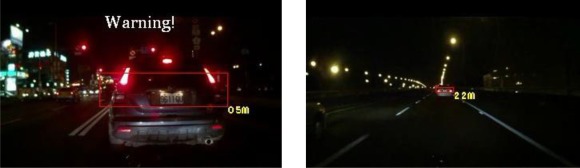
Results of vehicle detection and event determination for a nighttime urban road under bright illumination and free-flowing traffic conditions (Test video 6).

Figures 20–22 (Test videos 4–6) depict experimental video scenes of urban roads under different illumination conditions with congested and free-flowing traffic conditions. As Figures 20–22 illustrate, the proposed system successfully detects the target oncoming and preceding vehicles with their distances to the host car, despite interference from numerous non-vehicle illumination objects in various urban road environments with different ambient lighting conditions. The system also activates warning and event recording processes under various environmental conditions associated with the complexity of road scenes.

**Figure 23.**
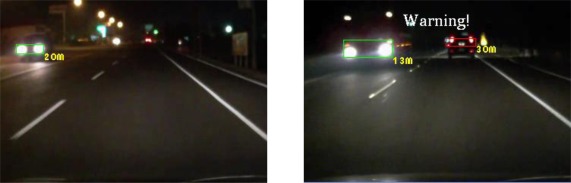
Results of vehicle detection and event determination for a nighttime highway under dim illumination and free-flowing traffic conditions (Test video 7).

**Figure 24.**
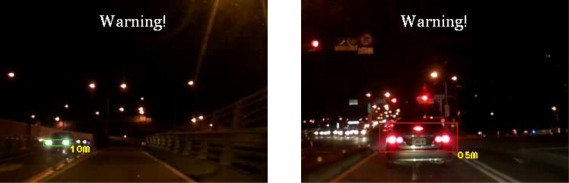
Results of vehicle detection and event determination for a nighttime highway under normal illumination and slightly congested traffic conditions (Test video 8).

**Figure 25.**
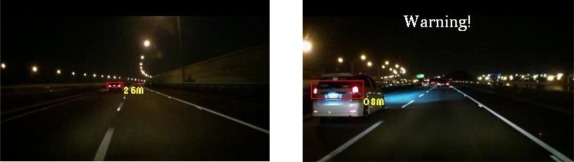
Results of vehicle detection and event determination for a nighttime highway under normal illumination and free-flowing traffic conditions (Test video 9).

Figures 23–25 (Test videos 7–9) show further experimental videos of highway environments under dim and normal illumination conditions with different traffic conditions. The snapshots in these figures demonstrate that the proposed system can also correctly detect oncoming and preceding vehicles, estimate their distances, and activate warning and event recording processes. In these three samples, when the oncoming vehicles appear and are detected by the proposed system, the ETDS module switches the headlights to low beam to avoid dazzling oncoming drivers. The ETDS modules then switches the headlights back to high beam after the oncoming vehicles have passed the host car.

To quantitatively evaluate vehicle detection performance, this study applies the Jaccard coefficient [39], which is commonly used to evaluate information retrieval performance. This measure is defined as:

(18) $$J=\frac{T_{p}}{T_{p}+F_{p}+F_{n}}$$

where *T_p_* (true positives) represents the number of correctly detected vehicles, *F_p_* (false positives) represents the number of falsely detected vehicles, and *T_n_* (false negatives) is the number of missed vehicles. The Jaccard coefficient *J* for the vehicle detection results of each frame of the traffic video sequences was calculated by manually counting the number of correctly detected vehicles, falsely detected vehicles, and missed vehicles in each frame. The average value of the Jaccard coefficients *J*, which serves as the detection score, was then obtained from all frames of the video sequences by:

(19) $$\bar{J}=\sum_{N}J/N$$

where *N* is the total number of video frames. The ground-truth of detected vehicles was obtained by manual counting. In the nine experimental videos shown sequentially from Figures 17–25, the numbers of preceding vehicles and oncoming vehicles appearing in the view of the experimental host car were 203 and 130, respectively. Table 1 shows the quantitative experimental results of the proposed system on vehicle detection performance. The performance of vehicle detection is relatively promising because the overall detection score reaches 92.08% in real nighttime road environments under various lighting conditions and complex traffic scenes.

In addition to quantitatively evaluating the vehicle detection performance of the proposed system, this study evaluates the detection accuracy of possible collision warning events of the ETDS module. The proposed system issues timely warnings to the driver to slow to avoid collision dangers, and automatically activates the traffic event recording process. Table 2 shows the quantitative detection accuracy data of the possible collision warning events of the proposed system. This table shows that the proposed system can achieve high detection accuracy of warning condition determination. The average event detection accuracy reaches 93.3% for different nighttime road environments with various lighting conditions and traffic conditions. Accordingly, the high detection accuracy on warning events demonstrates that the proposed system enables timely collision avoidance, and efficient storage usage for traffic event data recording.

For computational timing issues, the computation time required to process one input frame depends on the complexity of the traffic environment. Most of the computation time is spent on the spatial clustering process (including the connected component analysis) of potential lighting objects. Based on a TI OMAP3530 ARM-DSP embedded platform, the vision computing modules of the proposed VIDASS system require on average 70 milliseconds to process a video frame measuring 720 × 480 pixels, whereas the traffic event video recording takes approximately 10 ms per frame with hardware acceleration. The computational timing data of the vision processing modules are listed in Table 3. This computation cost ensures that the proposed system can effectively satisfy the demand of real-time processing at 12 fps (frames per second). Thus, the proposed system provides timely warnings and assistance for drivers to avoid possible traffic accidents.

## 5. Conclusions

This study presents a vision-based intelligent nighttime driver assistance and surveillance system (VIDASS system). The framework of the proposed system consists of a set of embedded software components and modules, and these components and modules are integrated into a component-based system on an embedded heterogamous multi-core platform. This study also proposes computer vision techniques for recording road-scene frames in front of the host car using a mounted CCD camera, and implement these vision-based techniques as embedded software components and modules. Vision-based technologies have been integrated and implemented on an ARM-DSP multi-core embedded platform and peripheral devices, including image grabbing devices and mobile communication modules. These and other in-vehicle control devices are also integrated to accomplish an in-vehicle embedded vision-based nighttime driver assistance and surveillance system. Experimental results demonstrate that the proposed system is effective and offers advantages for integrated vehicle detection, collision warning, and traffic event recording to improve driver surveillance in various nighttime road environments and traffic conditions. In the further studies, a feature-based vehicle tracking will be developed to more effectively detect and process the on-road vehicles having different number of vehicle lights, such as motorbikes. Besides, the proposed driver assistance system can also be improved and extended by integrating some sophisticated machine learning techniques, such as Support Vector Machine (SVM) classifiers, on multiple cues including vehicle lights and bodies, to further enhance the detection feasibility under difficult weather conditions (such as rainy and dusky conditions), and strengthen the classification capability on more comprehensive vehicle types, such as sedans, buses, trucks, lorries, small and heavy motorbikes.

## Figures and Tables

**Table 1.** Quantitative experimental data of the proposed system on detecting preceding and oncoming vehicles.

**Test videos**	**Detection Score J of preceding vehicles**	**Detection Score J of oncoming vehicles**

Test video 1 (Figure 17)	91.6%	100%
Test video 2 (Figure 18)	92.6%	N/A
Test video 3 (Figure 19)	93.8%	N/A
Test video 4 (Figure 20)	90.9%	88.6%
Test video 5 (Figure 21)	93.0%	84.6%
Test video 6 (Figure 22)	90.9%	92.6%
Test video 7 (Figure 23)	92.8%	82.5%
Test video 8 (Figure 24)	92.3%	83.1%
Test video 9 (Figure 25)	94.4%	N/A

Total no. of preceding vehicles	203	

Total no. of oncoming vehicles	130	

**Table 2.** Detection accuracy of the proposed system on warning event determination.

**Test videos**	**No. of actual warning conditions**	**No. of correctly determined warning conditions**	**Detection accuracy rate of warning events**

Test video 1	8	7	87.5%
Test video 2	15	14	93.3%
Test video 3	17	16	94.1%
Test video 4	8	8	100%
Test video 5	19	17	89.4%
Test video 6	16	15	93.8%
Test video 7	10	9	90%
Test video 8	15	15	100%
Test video 9	12	11	91.7%

Total no. of actual warning conditions	120		

No. of correctly determined warning conditions	112		

Average event determination accuracy	93.3%		

**Table 3.** The computational timing data of the vision processing modules of the proposed VIDASS system.

**Processing modules**	**Average computational times (ms per frame)**

Bright object segmentation module	8.12
Spatial analysis and clustering process module	45.03
Vehicle tracking and identification module	12.41
Vehicle distance estimation module	3.77
Event-driven traffic data recording subsystem	10.36
Total computational times	79.7
